# The origins of repetitive thought in rumination: Separating cognitive style from deficits in inhibitory control over memory

**DOI:** 10.1016/j.jbtep.2014.10.009

**Published:** 2015-06

**Authors:** Jonathan M. Fawcett, Roland G. Benoit, Pierre Gagnepain, Amna Salman, Savani Bartholdy, Caroline Bradley, Daniel K.-Y. Chan, Ayesha Roche, Chris R. Brewin, Michael C. Anderson

**Affiliations:** a MRC Cognition and Brain Sciences Unit, Cambridge, UK; b Harvard University, Department of Psychology, Cambridge, MA 02138, USA; c INSERM, U1077, 14033 Caen, France; d Université de Caen Basse-Normandie, UMR-S1077, 14033 Caen, France; e Ecole Pratique des Hautes Etudes, UMR-S1077, 14033 Caen, France; f Centre Hospitalier Universitaire, U1077, 14033 Caen, France; g University College London, London, UK

**Keywords:** Rumination, Retrieval suppression, Think/no-think, Inhibition, Memory

## Abstract

**Background and objectives:**

Rumination is a major contributor to the maintenance of affective disorders and has been linked to memory control deficits. However, ruminators often report intentionally engaging in repetitive thought due to its perceived benefits. Deliberate re-processing may lead to the appearance of a memory control deficit that is better explained as a difference in cognitive style.

**Methods:**

Ninety-six undergraduate students volunteered to take part in a direct-suppression variant of the Think/No-Think paradigm after which they completed self-report measures of rumination and the degree to which they deliberately re-processed the to-be-suppressed items.

**Results:**

We demonstrate a relation between rumination and impaired suppression-induced forgetting. This relation is robust even when controlling for deliberate re-processing of the to-be-suppressed items, a behavior itself related to both rumination and suppression. Therefore, whereas conscious fixation on to-be-suppressed items reduced memory suppression, it did not fully account for the relation between rumination and memory suppression.

**Limitations:**

The current experiment employed a retrospective measure of deliberate re-processing in the context of an unscreened university sample; future research might therefore generalize our findings using an online measure of deliberate re-processing or within a clinical population.

**Conclusions:**

We provide evidence that deliberate re-processing accounts for some – but not all – of the relation between rumination and suppression-induced forgetting. The present findings, observed in a paradigm known to engage top-down inhibitory modulation of mnemonic processing, provide the most theoretically focused evidence to date for the existence of a memory control deficit in rumination.

## Highlights

• Rumination has been linked to memory control deficits for emotional stimuli.
• Past work has not controlled deliberate reprocessing of to-be-suppressed memories.
• We demonstrate a link between rumination and impaired control for neutral memories.
• This relation remains when controlling for deliberate reprocessing.
• We provide strong evidence for a general memory control deficit in rumination.

## 1 Introduction

Cognitive control plays an important role in maintaining good mental health. For example, it allows us to direct attention away from thoughts that might otherwise upset us, and focus instead on more productive activities. However, when such control fails, we may instead find ourselves doing the opposite: Dwelling on negative thoughts, sometimes with dire consequences (although, see Andrews & Thompson, 2009).^1^ The tendency to perseverate on past negative experiences has been termed ‘depressive rumination’ (Nolen-Hoeksema, 1991, 2000) and has been found to predict the development of affective symptomatology such as suicidal ideation (e.g., Surrence, Miranda, Marroquin, & Chan, 2009). More recently, rumination has been recognized as a transdiagnostic process that is present in many anxiety and affective disorders (Ehring, Kleim, & Ehlers, 2011; McLaughlin & Nolen-Hoeksema, 2011), and appears to have a causal impact on the development of intrusive memories (Ball & Brewin, 2012). For this reason, it is perhaps no surprise that there has been growing interest in the cognitive and biological factors that predispose certain individuals towards rumination (for reviews, see Joormann, 2010; Whitmer & Gotlib, 2013).

In recent years, rumination has been linked to meta-cognitive beliefs concerning the utility and uncontrollability of repetitive thought (Papageorgiou & Wells, 2003). Whereas the belief that rumination is an adaptive cognitive strategy predicts the onset of rumination, the belief that rumination is uncontrollable or related to poor interpersonal or social outcomes has been found to mediate the relationship between rumination and depressive symptomatology. Perhaps lending credibility to beliefs concerning its uncontrollability, rumination has also been associated with executive dysfunction across a range of cognitive tasks, even after controlling for depression (e.g., De Lissnyder, Derakshan, De Raedt, & Koster, 2011; Joormann, 2005; Nolen-Hoeksema, Wisco, & Lyubomirsky, 2008; Whitmer & Banich, 2007). For example, rumination has been found to predict impairments in the ability to disengage attention in an antisaccade task (De Lissnyder et al., 2011) and also in the ability to inhibit previous task sets in a task-switching paradigm using either emotional or non-emotional materials (De Lissnyder, Koster, Derakshan, & De Raedt, 2010; Whitmer & Banich, 2007). Such dysfunction has been shown to precede (as opposed to follow) the onset of rumination, suggesting a critical role in the emergence of this behaviour (De Lissnyder et al., 2012; Whitmer & Gotlib, 2012). In fact, some theorists have argued that it is a general impairment in the ability to disengage attention from distracting or unwanted information that predisposes certain individuals towards ruminating in the first place (Koster, De Lissnyder, Derakshan, & De Raedt, 2011). Accordingly, the ability to focus on relevant information and suppress irrelevant information – either internally or externally – is critical in avoiding repetitive thought cycles, and “… individuals who are characterized by a difficulty to exercise attentional control in response to negative thoughts are likely to experience persistent rumination” (p. 139, Koster et al., 2011). Despite some accounts (e.g., Joormann, 2010), these impairments do not appear to be limited to emotional material – at least in the absence of depression (Whitmer & Gotlib, 2013).

### 1.1 Rumination and memory suppression

The clear linkage between rumination and inhibitory control deficits in attention tasks (for reviews, see Koster et al., 2011; Whitmer & Gotlib, 2013) raises the possibility that such deficits extend to disordered control over thoughts and memories. Although persistent thoughts and unwanted memories may appear to differ in many ways, most psychological disorders are characterized by attempts to avoid both (Brewin, Gregory, Lipton, & Burgess, 2010). Moreover, the two often occur simultaneously and are reciprocally connected (Newby & Moulds, 2012; Pearson, Brewin, Rhodes, & McCarron, 2008). Thus the notion that individual differences in the efficacy of cognitive control could predict ruminative tendencies has also led to work addressing the link between rumination and memory suppression. Memory suppression refers to the ability to suppress retrieval of an unwanted memory when faced with a reminder and is typically measured using the think/no-think (TNT) paradigm. In this paradigm, participants learn cue-target word pairs until the cue reliably activates the associated target. They then undergo a series of trials in which a subset of the studied cue words is sequentially presented and participants must either retrieve (Think trials) or suppress (No-Think trials) the associated target word. This process is repeated multiple times for each cue word, resulting in some target words that are repeatedly retrieved and others that are repeatedly suppressed. The typical finding is that memory for the retrieved (Think, or Respond) items is significantly *better* than memory for baseline items that were neither retrieved nor suppressed (the *positive control effect*) whereas memory for the suppressed (No-Think, or Suppress) items is significantly *worse* than memory for baseline items that were neither retrieved nor suppressed (the *negative control effect*, or *suppression-induced forgetting*). These effects are robust as demonstrated in cued-recall (e.g., Anderson & Green, 2001), recognition memory (e.g., Waldhauser Lindgren, & Johansson, 2012) and indirect memory measures (e.g., Gagnepain, Henson, & Anderson, 2014). Suppression is also evident in neural indices of implicit memory such as neural priming (Gagnepain et al., 2014). Importantly, the effects of retrieval suppression arise even when an independent probe cues retrieval instead of the original cue with which the target item was studied (e.g., Anderson & Huddleston, 2012). The cue independence of suppression-induced forgetting excludes interference as a possible explanation, establishing the role of inhibition in producing the phenomenon (Huddleston & Anderson, 2012).

We propose that memory suppression as measured using the TNT paradigm reflects the action of the same underlying control processes required when mitigating the intrusions associated with a ruminative thought: just as the retrieval of a no-think target must be suppressed when it intrudes in response to its cue word, an unwanted thought or memory concerning a negative event must likewise be controlled using similar processes, lest it perseverate in awareness and re-emerge in response to reminders. Suppression in the TNT paradigm substantially reduces the frequency of intrusive memories with repetition, purging intrusions from awareness via inhibitory control mechanisms that down-regulate hippocampal activity (Benoit, Hulbert, Huddleston, & Anderson, 2014; Levy & Anderson, 2012). If so, the TNT paradigm represents a theoretically focused means of evaluating the executive deficits thought to predispose individuals towards symptomatology such as rumination – with the prediction that ruminative tendencies should be associated with impaired suppression and therefore reduced suppression-induced forgetting (Levy & Anderson, 2008). Two studies already support this hypothesis. First, Hertel and Gerstle (2003) used the TNT paradigm with positively or negatively valenced word pairs to measure suppression in dysphoric and nondysphoric populations. They found that rumination was associated with both (a) greater recall of the no-think items, and (b) a smaller difference in recall between think and no-think items. This finding remained even when accounting for dysphoria. Dieler, Herrmann, and Fallgatter (2014) extended these findings. Using pairs of neutral faces and either negative or neutral target images, they demonstrated a correlation between ruminative brooding and reduced suppression-induced forgetting, but only for negative targets.

### 1.2 The current experiment

The negative correlation observed between rumination and suppression-induced forgetting is consistent with the theoretical argument that impaired control processes predispose individuals towards ruminative tendencies. However, this relationship may not reflect an inability to implement control, but rather a tendency not to do so. For example, whereas impaired memory suppression might predispose individuals towards rumination, we speculate that ruminative individuals may also simply choose not to follow the experimental instructions to suppress retrieval as closely as do non-ruminative individuals. Highly ruminative individuals often believe that repetitive thought is valuable (e.g., Papageorgiou & Wells, 2003), raising the possibility that earlier investigations may simply have conflated deficits in suppression-induced forgetting with differences in cognitive style. In a recent review, Anderson and Huddleston (2012) argued that variation in suppression-induced forgetting could be attributable in part to differences in the degree to which participants possessed either the capacity or the willingness to suppress retrieval of the no-think items. By this view, the capacity to suppress retrieval relates to the efficacy of inhibitory control, and so factors thought to influence inhibitory control should mitigate retrieval suppression and its behavioral consequences. However, even a substantial inhibitory capacity accomplishes little if participants are not disposed to suppress, and instead deliberately re-process the no-think items. People disposed towards rumination may have a habit of revisiting thoughts voluntarily, which would undo the effects of suppression, a finding that could masquerade as a deficit in inhibitory control over memory.

The current investigation examines whether the relation between rumination and memory suppression remains after accounting for ruminators' tendency to voluntarily re-engage to-be-suppressed thoughts. In so doing, we seek to better evaluate whether rumination is truly associated with diminished mnemonic control. If rumination represents a particular response style independent of any control deficits, those exhibiting chronic rumination may be more inclined to intentionally reflect on their memory for the no-think items. If this hypothesis is correct, rumination scores should predict participants’ tendency to violate our suppression instructions by deliberately re-processing no-think items both during and after the trials on which they are meant to be suppressing retrieval. To the extent that measures of deliberate re-processing represent conscious, intentional retrieval of no-think items, we can use this measured noncompliance to ascertain whether the predicted relationship between rumination and memory suppression survives when variability due to voluntary re-processing is controlled statistically.

To address this question, we used the think/no-think paradigm (based upon Anderson & Green, 2001) with neutral word-pairs. We selected neutral stimuli so that we can clearly establish that any observed deficit reflects a general difficulty with inhibitory control over memory, and not a problem in disengaging from emotional material. Thus far, the relation between rumination and memory suppression, like the relation between rumination and directed forgetting (Joormann & Tran, 2009), has only been observed using emotional stimuli (Dieler et al., 2014; Hertel & Gerstle, 2003), and was not found to be present with neutral stimuli (Dieler et al., 2014). This finding is contrary to other studies reporting rumination-related cognitive control deficits that appear to be unaffected by valence, at least within non-depressed populations (see Whitmer & Gotlib, 2013).

Moreover, because we sought to quantify variation in an inhibitory process that we believe to be common to both memory suppression and the mitigation of repetitive thought, we needed to ensure that participants used a uniform strategy, and ensure that the inhibitory control process hypothesized to be deficient was likely to be engaged. We therefore used direct suppression task instructions (Benoit & Anderson, 2012; Bergstrom, de Fockert, & Richardson-Klavehn, 2009) instead of unguided suppression. Direct suppression instructions request that participants solve the No-Think task by suppressing *any effort to retrieve anything at all in response to the cue* – even distracting thoughts that might otherwise occupy awareness – and, moreover, to push the unwanted memory out of awareness in cases in which it does intrude. Direct suppression may be distinguished from thought substitution, which instead involves generating substitute memories or thoughts to distract oneself, and so actively engages the retrieval process. Although both direct suppression and thought substitution induce forgetting of No-Think items, the neural mechanisms underlying them have been dissociated: Whereas *direct suppression* involves the down-regulation of neocortical and/or hippocampal regions by the right dorsolateral prefrontal cortex, *thought substitution* instead recruits the left ventrolateral prefrontal cortex to aid in selecting an appropriate substitute (Benoit & Anderson, 2012; Gagnepain et al., 2014).

Because the distinction between direct suppression and thought substitution has only recently been investigated, the majority of the current literature (including those findings summarized above) has employed general, unguided task instructions that do not clearly favor either strategy. As a consequence, much of the current data on suppression-induced forgetting may reflect an aggregation of the neural processes summarized above. Therefore, the second motivation of the current project was to further demonstrate suppression-induced forgetting in a large sample of participants explicitly instructed to employ a direct suppression strategy (e.g., Benoit & Anderson, 2012). By demonstrating suppression-induced forgetting under circumstances in which participants are asked to fully suppress retrieval (as opposed to generate substitutes), we expect our measure to be more likely to reflect the action of the fronto-hippocampal modulatory mechanism believed to underlie voluntary memory inhibition, and so provide a more theoretically focused test of the role of memory inhibition deficits in rumination.

## 2 Material and methods

### 2.1 Participants

A sample of 114 undergraduate students from University College London participated in exchange for course credit. Of this sample, 96 (41 males, 55 females) completed the rumination measure; only this sub-sample was included in our primary analyses. Participants were recruited using online advertisements and all procedures and materials were approved through the Ethics Committee of the University College London Division of Psychology and Language Sciences. Written informed consent was obtained.

### 2.2 Stimuli and apparatus

Stimuli consisted of 36 experimental word pairs and 18 filler pairs. All pairs were neutral in valence. The experimental word pairs were counterbalanced across condition (think, no-think and baseline). Filler word pairs served as buffers at the beginning and end of the learning and test phases to minimize primacy and recency effects and also as practice items during the think/no-think phase. Performance was not analyzed for filler items.

### 2.3 Procedure

**Fig. 1:**
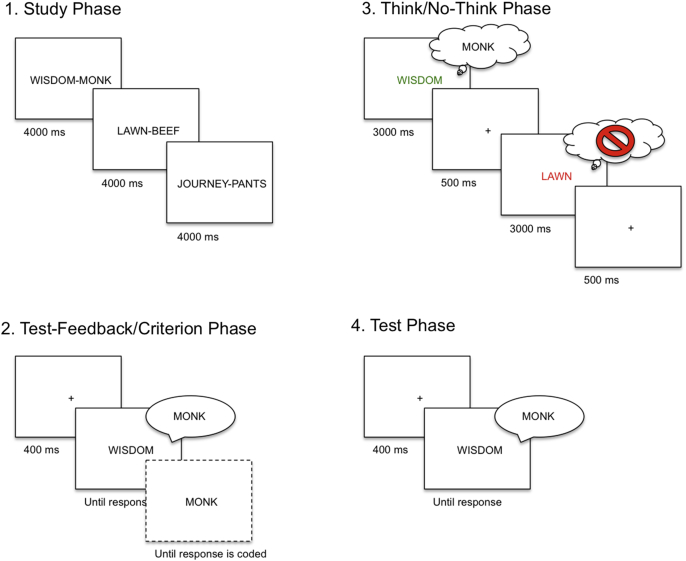
Participants first learned the word pairs in the study phase, after which they practiced retrieving the target word aloud when presented with the cue for each pair. Once participants either completed this cycle twice or reached at least 50% performance in the test-feedback phase, they completed one final criterion test that was identical with the exception that no feedback was presented (as represented by the dotted line in the figure). Participants then completed the Think/No-Think (TNT) phase. For Think items (in green), participants retrieved the associated target. For No-Think items (in red), they were asked to prevent the target from coming to mind without distracting themselves with other thoughts. Following the TNT phase participants completed the test phase, in which they were presented with each hint word and were instructed to recall the corresponding target aloud. (For interpretation of the references to colour in this figure legend, the reader is referred to the web version of this article.)

The procedure for this experiment is depicted in Fig. 1.

#### 2.3.1 Study phase

In the initial portion of the study phase, participants studied each word pair for 4 s after which they practiced retrieving the target word when presented with the cue for each pair. During this portion of the phase, each cue appeared one at a time in the center of the screen (preceded by a 400 ms fixation) and remained until participants produced the associate out loud. The correct target was presented 400 ms later and remained onscreen until the experimenter pressed a button to code for response accuracy. Participants were instructed to use the appearance of the target as an opportunity to better learn the cue-target pairs. This process was continued until a threshold of 50% accuracy was achieved or until the phase was repeated thrice. Participants were then given one final criterion test without feedback to gauge whether they learned each item.

#### 2.3.2 Think/No-think phase

During the think/no-think phase, participants were presented with two-thirds of the studied cue words, one at a time, in either green or red font. For the green words, participants were asked to covertly recall and maintain the relevant target word (Think condition). For red words, participants were instead instructed to focus on the cue while clearing their mind of all other thoughts, especially the target word (No-Think condition). During these trials they were also instructed to never replace the target word with any other thought or word. These instructions correspond to the direct suppression instructions used by past researchers (e.g., Benoit & Anderson, 2012; Bergstrom et al., 2009; Gagnepain et al., 2014). Each cue word was presented for 3 s, after which a fixation cross appeared for 500 ms. Before the experimental think/no-think trials began, participants completed 35 practice trials using the filler items split into two short phases. A break occurred halfway through the practice phase during which the experimenter administered a brief questionnaire and offered feedback on how participants were performing the task. Overall, participants completed a total of 288 think/no-think trials representing 12 repetitions of each item. Trials were split into six blocks with two repetitions of each item within each block. The fixed presentation order for each block was created pseudo-randomly with the constraints that (i) there were no more than 3 consecutive cues of the same color, and (ii) a given cue could only be repeated once all other cues had been presented within a block.

#### 2.3.3 Test phase

Participants were once again tested for all word pairs in a manner identical to the final criterion test of the study phase. On each trial, a single cue word was presented for 4 s followed by a fixation cross that was presented for 400 ms. Participants were instructed to recall the relevant target word out loud. No feedback was given and responses were recorded and scored by the researcher.

#### 2.3.4 Post-experimental questionnaires

Following the test phase, participants completed the 22-item Ruminative Responses Scale (RRS; Treynor, Gonzalez, & Nolen-Hoeksema, 2003). Past studies have found the ruminative responses scale to demonstrate high internal reliability (Cronbach alpha ≈0.90; for a review, see Luminet, 2004); this measure is also thought to estimate stable ruminative tendencies, as evidenced by high test-retest reliability (*r* ≈ 0.80; Nolen-Hoeksema, Parker, & Larson, 1994; Nolen-Hoeksema & Davis, 1999) even across a period greater than 1.5 years. Our sample mean for this measure was 42.74 (*SE* = 1.00) with scores ranging from 24 to 75 and with first and third quartiles of 35 and 49, respectively. Cronbach's alpha as calculated in our sample was suitably high and similar to those reported in past studies (*α* = .88).

In addition, participants completed two self-report questionnaires measuring (a) the strategies employed during the Think/No-Think phase, and, (b) the extent to which they deliberately re-processed the to-be-suppressed items during no-think trials, against our instructions. During the strategy questionnaire participants rated how often they used strategies related to either direct suppression (e.g., *stared intently at the red word*, *kept mind clear*) or thought substitution (e.g., *generated a related thought or idea*, *generated a memory*) on a scale from *Never* (0) to *Always* (4). Direct suppression strategies (*M* = 1.92, *SE* = 0.07) were significantly more common than thought substitution (*M* = 0.68, *SE* = 0.06), *t*(92) = 13.93, *p* < .001, *d* = 2.90. This represents a substantial reduction in the use of thought substitution as a strategy relative to samples in which direct suppression instructions were not given (e.g., typical thought substitution scores range between 2.25 and 2.75; e.g., see also, Levy & Anderson, 2008, Figure 5 for strategy percentages in a large uninstructed sample) and suggests compliance with this aspect of our task instructions.

The re-processing measure was comprised of two key self-report items in which participants indicated how frequently (from never [0] to very frequently [4]) they (a) reflected briefly about the target word during no-think trials, prior to suppressing awareness and (b) revisited briefly their thoughts about the target word after each no-think trial was over, and the cue word was removed from the screen.^2^ Responses were summed to form a deliberate re-processing score ranging from 0 to 8. Our sample mean for this measure was 1.13 (*SE* = 0.12) with scores ranging from 0 to 5 and with first and third quartiles of 0 and 2, respectively.

## 3 Results

Analyses were conducted on recall scores conditionalized on initial learning performance (e.g., Anderson et al., 2004). Thus, items were only included if they were recalled during the criterion test at the end of the learning phase. Because unlearned items can be neither suppressed nor retrieved during the Think/No-Think phase, they should contribute only noise to the data, mitigating any statistical effects, justifying their exclusion. Prior to analysis, one participant was excluded as an extreme outlier for demonstrating a suppression-induced forgetting effect exceeding 4.5 standard deviations from the mean.

### 3.1 Basic analyses

**Fig. 2:**
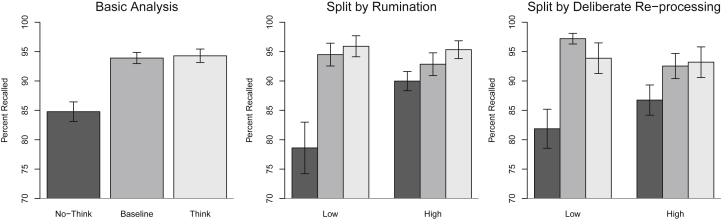
The percent recalled target items (conditionalized on initial learning performance) as a function of condition (No-Think, Baseline, Think) in the total sample and then separated into low/high groups according to rumination or deliberate re-processing scores. To maximize statistical power, rumination and deliberate re-processing were dichotomized to compare only the lower and upper quartiles (e.g., Gelman & Park, 2009): The “low” and “high” groups were defined as those individuals scoring in the bottom 25% and the top 25% of the relevant metric, respectively, with the middle 50% of the sample excluded. Error-bars for the left panel represent within-subject standard error (e.g., Franz & Loftus, 2012). All other error bars represent between-subject standard error with the pairwise comparisons reported in-text.

We first analyzed the effect of condition (Suppress, Baseline, Respond) using a repeated-measure ANOVA. As depicted in Fig. 2, the main effect of condition was significant, *F*(2, 188) = 21.62, *MSE* = 127.46, *p* < .001, η_g_^2^ = 0.110. Comparisons conducted using Fisher's Least Significant Difference (*LSD* = 3.23; Williams & Abdi, 2010) revealed performance to be lower for the Suppress items than for the Baseline items. Performance was similar for the Baseline and Respond items. Thus, suppression-induced forgetting (Baseline – Suppress = 9.13%, *SE* = 1.74%) was observed, whereas little benefit of repeated retrieval was found (Respond – Baseline = 0.38%, *SE* = 1.31%), perhaps because overall performance was high.^3^

### 3.2 Relation between suppression, rumination and deliberate Re-processing

We next considered the relation between the magnitude of suppression-induced forgetting (Baseline – Suppress) and self-reported rumination and deliberate re-processing scores. Suppression-induced forgetting was negatively correlated with both rumination, *r* = −0.25, *p* = .014, and deliberate re-processing, *r* = −0.22, *p* = .034. Although the positive control effect did not correlate with either rumination, *r* = 0.05, *p* = .640, or deliberate re-processing, *r* = 0.14, *p* = .166, these particular correlations must be interpreted cautiously due to the apparent absence of a positive control effect in our sample. As hypothesized based on prior literature, rumination scores did indeed predict deliberate re-processing during No-Think trials, *r* = 0.25, *p* = .014. This rumination/deliberate re-processing correlation complicates interpretation of the individual suppression-induced forgetting/rumination and suppression-induced forgetting/re-processing correlations due to shared variance between these predictors. To determine the origin of the observed effects, semi-partial correlations were calculated exploring the relation between suppression-induced forgetting and each variable, controlling for the remaining variable (Algina & Keselman, 2010): Put differently, the correlation between suppression-induced forgetting and rumination was calculated while controlling statistically for deliberate re-processing, and then the correlation between suppression-induced forgetting and deliberate re-processing was calculated while controlling statistically for rumination. The resulting metric measured the variance in suppression-induced forgetting explainable by rumination or deliberate re-processing, independent of each other. Whereas the semi-partial correlations revealed a significant relation between suppression-induced forgetting and rumination, *r* = −0.20, *p* = .045, the relation between forgetting and deliberate re-processing was in the predicted direction but was no longer significant, *r* = −0.16, *p* = .121.^4^ This outcome reinforces the relation between rumination and impaired suppression-induced forgetting, while suggesting that the relation between deliberate re-processing and impaired forgetting is also driven in part by ruminative tendencies.

To further illustrate the relationship between rumination/deliberate re-processing and memory suppression, supplementary quartile split analyses were also conducted for the rumination and re-processing comparisons (see right panel of Fig. 2). To maximize statistical power, each variable was dichotomized to compare only the lower and upper quartiles (e.g., Gelman & Park, 2009). As evident in Fig. 2, the low rumination group demonstrated significantly more suppression-induced forgetting (*M* = 0.16, *SE* = 0.03) than did the high rumination group (*M* = 0.03, *SE* = 0.03; *t*(46) = 3.05, *p* = .004); once again, practically no difference was observed between these groups for the magnitude of the positive control effect (Low: *M* = 0.01, *SE* = 0.01; High: *M* = 0.03, *SE* = 0.02; *t*(46) = 0.43, *p* = .666). Indeed, the differences in the amount of suppression-induced forgetting appeared to be driven exclusively by numerical differences in the recall of suppress items. The deliberate re-processing analysis revealed a similar pattern. The low re-processing group (*M* = 0.15 *SE* = 0.03) demonstrated significantly more suppression-induced forgetting than did the high re-processing group (*M* = 0.06, *SE* = 0.03; *t*(46) = 2.09, *p* = .042) but no difference in the facilitation effect (Low: *M* = −0.03, *SE* = 0.03; High: *M* = 0.01, *SE* = 0.02; *t*(46) = 0.95, *p* = .349). However, unlike the rumination analysis, the difference in suppression-induced forgetting for the deliberate re-processing analysis arose from the combination of numerically greater recall of suppress items and numerically lesser recall of baseline items in the high as compared to low re-processing groups.

## 4 Discussion

The current study demonstrates a clear relationship between self-reported rumination and memory suppression ability. Importantly, it is the first to measure this relationship while controlling statistically for differences in the tendency to voluntarily re-process the to-be-suppressed memories. Because deliberate re-processing is correlated with both rumination and memory suppression, failure to account for this behavior when considering rumination-related differences in memory suppression risks conflating inhibitory deficits with more general differences in processing style. Our findings demonstrate that even controlling for deliberate re-processing, rumination significantly predicts impaired suppression-induced forgetting. Further, we demonstrate that this relation is wholly attributable to greater memory for the suppress items; rumination does not predict memory for either the baseline or respond items. These findings complement recent evidence that self-reports of thought control ability in daily life predict suppression-induced forgetting of aversive scenes (Kuepper, Benoit, Dalgleish, & Anderson, 2014), and converge on the view that people are sensitive to their own capacity to regulate unwanted thoughts.

In the current procedure, we measured the tendency to deliberately re-process unwanted information by asking participants to rate the extent to which they intentionally thought about to-be-suppressed items, irrespective of our instructions not to. By controlling for this measured tendency for deliberate re-processing, the remaining correlation between rumination and suppression better isolates inhibitory impairments. Another interpretation, however, might be that deliberate re-processing instead simply captures whether participants become aware of unintentional intrusions. Ruminative individuals often report the belief that their repetitive thoughts are difficult to control (e.g., Papageorgiou & Wells, 2003). These meta-cognitive beliefs – which predict the development of depression and continuing repetitive thoughts (Papageorgiou & Wells, 2004) – might represent insight into their own deficits. By this interpretation, our measure of deliberate re-processing may not be distinct from an inhibitory deficit.

We do not favor this interpretation, however, because it assumes that participants interpret unintentional intrusions as intentional retrieval. We consider this unlikely. We purposely framed the questions on our measure to emphasize a deliberate, willful act, and included (a) an admonishment in the instructions (i.e., “Each of the following three statements is intended to measure whether you ever INTENTIONALLY made an effort to think about the responses for the red hint items”), and (b) a brief interaction with the participant to make sure that they were clear that we were not asking about accidental remindings, but purposeful reflection. Instead, we speculate that the greater incidence of deliberate re-processing in ruminative individuals reflects their habitual tendency to revisit thoughts, because they believe this is beneficial. Indeed, participants may reflect on the suppress items as a means of evaluating how effectively they were suppressed. This conceptualization is analogous to the finding that rumination in depression is related to beliefs concerning the benefits of repetitive thought (e.g., Papageorgiou & Wells, 2003). However, even if our measure of deliberate re-processing reflects some contribution of misinterpreted involuntary remindings, this would only strengthen the central theoretical conclusion that ruminators suffer from an inhibitory deficit, given that the relationship remained after accounting for this variable.

It is worth noting that current findings did not replicate the null result observed by Dieler et al. (2014) concerning the correlation between rumination and the suppression of emotionally neutral material. One possibility pertains to the fact that whereas Dieler et al. (2014) screened their participants for psychiatric disorders including depression, the current experiment did not. Because the consequences of rumination can at times vary between depressed and non-depressed populations (e.g., for discussion, see Whitmer & Gotlib, 2013), our present findings must be viewed with some degree of caution. However, even beyond this possibility, there exist several methodological differences that could readily account for the discrepancy between our studies. First, Dieler et al. (2014) employed an unusually long inter-trial interval during their think/no-think phase (a delay of ∼8.5 s between each 4 s trial, instead of 500 ms). An extended delay following no-think trials provides greater opportunity for intrusions (or deliberate re-processing) to occur, as is often reported by participants during these tasks. This may also explain why they failed to observe suppression-induced forgetting without including rumination in their model. Second, whereas this earlier study employed general Think/No-Think task instructions, we used direct suppression instructions that should provide a better measure of top-down inhibitory modulation over mnemonic processes (Benoit & Anderson, 2012; Gagnepain et al., 2014). The present findings therefore strongly reinforce the existence of a general memory control deficit in ruminative persons (e.g., Hertel & Gerstle, 2003), rather than a focused difficulty with controlling emotionally negative material, though it remains to be seen whether ruminators have even greater difficulty with inhibiting emotionally negative memories.

### 4.1 Limitations

One limitation of our experiment derives from the fact that the current sample was taken from an undergraduate population. Therefore, it is reasonable to wonder whether these conclusions would also apply to a clinical sample. However, since we did not screen out symptomatic participants, it would be incorrect to assume that our undergraduate sample is healthy. As noted by Ibrahim, Kelly, Adams, and Glazebrook (2013) in a meta-analysis of 24 studies of depression prevalence in university students, 30.6% of university undergraduates suffer from depression, a rate markedly higher than estimated in the general population. Consistent with this, our mean full-scale RRS score of 42.73 was 10 points higher than that reported for a healthy community sample defined as excluding any person meeting diagnostic criteria for any current or past Axis I disorder (e.g., RRS = 32.34: Joormann, Levens, & Gotlib, 2011). Thus, a similar study using a clinical sample would be useful as a future direction and could prove crucial in determining whether our findings apply equally to both healthy adults as well as those suffering from depression.

Another limitation derives from the fact that we used a retrospective as opposed to online measure of deliberate re-processing. Retrospective measures depend on intact memory for the processes at work during the Think/No-Think phase. The only alternative to doing this retrospectively, however, was to ask participants, on a trial-by-trial basis, whether they engaged in deliberate reprocessing. Although this would reduce variance due to retrospective memory failures, it would also undermine the suppression task itself, by prompting participants to engage in these proscribed behaviors. As such, we judged the retrospective approach to be the least disruptive way of assessing deliberate reprocessing. Nevertheless, it would be useful to see whether measuring deliberate reprocessing online would more firmly establish a relation between this behavior and suppression-induced forgetting, independent of inhibition deficits.

## 5 Conclusions

The present findings provide some of the strongest evidence to date that rumination is associated with diminished inhibitory control over memory and that this impairment could underlie the core deficit. Our findings are particularly diagnostic because they are the first to control for differences in cognitive style between ruminators and non-ruminators, to demonstrate this relation in the context of non-emotional stimuli and to use direct suppression instructions that recent neuroimaging evidence suggests better isolate top-down inhibitory modulation of mnemonic processing (e.g., Benoit & Anderson, 2012). We believe that our findings support a memory control deficit associated with rumination that is dissociable from a general cognitive style that might likewise encourage repetitive thought.

An important implication is that clinical interventions applied to ruminative populations should address both the maladaptive beliefs concerning repetitive thought and also the apparent inhibitory deficits that might complicate the suppression of those thoughts once rumination has begun. For example, a recent intervention aimed specifically at rumination in treatment-resistant depression included elements designed to improve attentional control as well as modifying meta-cognitive beliefs (Wells et al., 2012). Other promising approaches that have brought about significant reductions in depressive rumination have sought to modify the intrusion of unwanted memories by having depressed patients vividly imagine positive competitor memories, and if necessary re-scripting negative memories (Brewin et al., 2009; Ekkers et al., 2011). At present it is not known if these interventions operate by enhancing general inhibitory control. Designing more specific interventions targeted at inhibitory processes, as well as identifying possible genetic contributions to inhibitory control, holds promise for maximizing the effectiveness of therapies for rumination.

## Footnotes

1 It is worth noting that whereas rumination is generally viewed negatively, this is not universally true: Rumination is sometimes viewed as an adaptive process aimed at ameliorating an aversive environmental or emotional challenge. For example, Andrews and Thompson (2009) have argued that brain regions affiliated with sustained attention (e.g., the left ventrolateral prefrontal cortex) become more active during depression. Viewed from this light, rumination could reflect an emergent property of a neurobiological mechanism that encourages a fixation on current problems and could – perhaps with the help of psychotherapy – lead to resolution. Because the current experiments deal with the control of rumination rather than its adaptive potential, we do not address this possibility further, but rather direct the interested reader to Andrews and Thompson (2009).

2 A third item typically included in this questionnaire (During No-Think trials, I thought about the item that went with the hint word to improve my memory for that item) was excluded on the basis that it did not reflect re-processing in the same manner as the other two. It had more to do with intentional memorization. This decision was made prior to analysis, and inclusion of that question does not impact the nature or significance of any of the reported findings.

3 A similar pattern was observed in the full sample of 113 participants, *F*(2, 224) = 21.90, *MSE* = 116.76, *p* < .001, η_g_^2^ = 0.095, *LSD* = 2.83. Performance was lower for the Suppress items (*M* = 86.13%, *SE* = 1.47%) than for Baseline items (*M* = 94.37%, *SE* = 0.82%) or Respond items (*M* = 94.36%, *SE* = 1.02%).

4 Semi-partial correlations exclude common variance between one variable in a correlation (e.g., rumination) and some other controlled variable (e.g., deliberate reprocessing) while preserving the variance of the remaining variable in the correlation (the suppression-induced forgetting effect). Another approach is to exclude common variance between each variable in the correlation and the controlled variable, resulting in a partial correlation (Delaney, 2010). Partial correlations are always larger than semi-partial correlations but in this case produce the same pattern: A significant relation between suppression-induced forgetting and rumination, *r* = −0.21, *p* = .040, the relation between forgetting and deliberate re-processing was in the predicted direction but was no longer significant, *r* = −0.16, *p* = .109. Non-parametric alternatives were also calculated for all basic, partial and semi-partial correlation analyses (i.e., Kendall's Tau and non-parametric bootstrapped regression models) and the same effects emerged. We have reported Pearson correlations instead for ease of interpretation.
